# Evaluation of Lower-Limb Kinematics during Timed Up and Go (TUG) Test in Subjects with Locomotive Syndrome (LS) Using Wearable Gait Sensors (H-Gait System)

**DOI:** 10.3390/s23020687

**Published:** 2023-01-06

**Authors:** Yoshiaki Kataoka, Yuki Saito, Ryo Takeda, Tomoya Ishida, Shigeru Tadano, Teppei Suzuki, Kentaro Nakamura, Akimi Nakata, Satoshi Osuka, Satoshi Yamada, Mina Samukawa, Harukazu Tohyama

**Affiliations:** 1 Faculty of Health Sciences, Hokkaido University, Sapporo 060-0812, Japan; 2 Department of Rehabilitation, Health Sciences University of Hokkaido Hospital, Sapporo 002-8072, Japan; 3 Faculty of Engineering, Hokkaido University, Sapporo 060-8628, Japan; 4 Iwamizawa Campus Midorigaoka, Hokkaido University of Education, 2-34, Iwamizawa 068-864, Japan

**Keywords:** timed up and go test, wearable sensor, locomotive syndrome, kinematics

## Abstract

Few studies have dealt with lower-limb kinematics during the timed up and go (TUG) test in subjects with locomotive syndrome (LS). This study aimed to evaluate the characteristics of lower-limb kinematics during the TUG test in subjects with LS using the wearable sensor-based H-Gait system. A total of 140 participants were divided into the non-LS (*n* = 28), the LS-stage 1 (*n* = 78), and LS-stage 2 (*n* = 34) groups based on the LS risk test. Compared with the non-LS group, the LS-stage 1 and LS-stage 2 groups showed significantly smaller angular velocity of hip and knee extension during the sit-to-stand phase. The LS-stage 2 group showed significantly smaller peak angles of hip extension and flexion during the walking-out phase compared to the non-LS group. These findings indicate that the evaluation of the lower-limb kinematics during the TUG test using the H-Gait system is highly sensitive to detect LS, compared with the evaluation of the lower-limb kinematics when simply walking.

## 1. Introduction

With the rapid aging of the population in Japan, a prevention strategy for locomotive organ disorders is urgently required. The Japanese Orthopaedic Association (JOA) proposed the term “locomotive syndrome (LS)” for a condition in which motor function is impaired due to disorders of the locomotive system consisting of bones, joints, muscles, and nerves requiring or potentially requiring nursing care [1]. The weakness of the locomotive component causes difficulty in mobility, which is an essential physical function for daily living, such as standing or walking. Recent clinical studies show that approximately 47 million people in Japan are diagnosed with osteoarthritis of the knee, spondylolisthesis, or osteoporosis on radiographs and are at risk for LS [2]. The JOA established the LS risk test as a diagnostic criterion [3,4], which classifies the risk level to be 1 or 2 [5]. However, since the LS risk test is a semi-quantitative and comprehensive assessment, a more detailed assessment was needed to evaluate walking ability in detail to prevent LS.

Gait analysis is used in many clinical settings to diagnose disabilities and evaluate walking ability. Recently, we have developed a wearable sensor-based system (H-Gait) that utilizes seven wearable sensors consisting of tri-axial acceleration sensors and tri-axial gyro sensors to analyze the lower-limb kinematics during walking without optical tracking [6]. This system can calculate lower-limb kinematics while walking easily and quickly without the restriction of the location [7,8]. Moreover, we revealed the characteristics of lower-limb kinematics during the 10 m walking test using the H-Gait system in subjects with LS [9]. This study detected differences in lower-limb kinematics during the 10 m walking test between subjects with non-LS and subjects with LS-stage 2, but not between non-LS and LS-stage 1 subjects. Therefore, it is necessary to investigate other gait function assessment methods to detect differences in lower-limb kinematics between non-LS and LS-stage 1 subjects, such as the timed up and go (TUG) test, which can assess the walking ability of the elderly in more detail. Nishizawa et al. also mentioned the need for the evaluation of kinematics using this test [10]. However, few studies dealt with lower-limb kinematics during the TUG test in subjects with LS.

The TUG test consists of movements essential for daily living, such as standing up from a chair, walking, changing direction, and sitting on a chair and is a quick and simple method for assessing lower-limb function, mobility, and fall risk [11,12]. TUG completion time is excellent for evaluating waking ability and gait function, and the subjects with LS have been shown to have a longer TUG completion time than the non-LS subjects [13], with a strong positive correlation between LS severity and TUG completion time [14]. However, the measurement of TUG completion time alone may not be able to provide a detailed evaluation of the loss of walking ability and gait function. The evaluation of movement characteristics during the TUG test using inertial sensors, rather than only completion time, can detect the loss of walking ability [15]. Although an optical motion analysis system using a camera has been generally used to analyze lower-limb kinematics during the TUG test, it imposes limitations on the time and location of the measurement [16]. In recent years, due to technological advances, motion analysis using acceleration and gyro sensors has been verified and clinically applied to gait analysis during the TUG test [17,18,19,20,21]. The H-Gait system can also make it possible to evaluate lower-limb kinematics during the TUG test. Therefore, this study aimed to evaluate the characteristics of lower-limb kinematics during the TUG test in subjects with LS using the H-Gait system. In particular, we aimed to detect differences in lower-limb kinematics during the TUG test between non-LS and LS-stage 1 subjects, as previous studies failed to detect differences during the 10 m walking test [9]. We hypothesized that the lower-limb kinematics during the TUG test differed between non-LS and LS-stage 1 subjects.

## 2. Materials and Methods

### 2.1. Participants

The participants were recruited from local residents who used a public health promotion facility in Iwamizawa city, Japan. A total of 140 individuals participated in this study (22 men and 118 women: age 72.6 ± 6.7 years, height 152.7 ± 7.3 cm, and weight 53.4 ± 8.4 kg). The participants were included if they were (1) aged 60 years or above and (2) able to complete the TUG test and the LS risk test independently without a walking aid. The exclusion criteria were individuals who had any acute or uncontrolled cardiac, pulmonary, or musculoskeletal conditions, severe visual impairment, or cognitive impairment. This study was approved by the institutional review board of our university (#18-50), and the participants were informed that data from this study would be submitted for publication and gave their consent.

### 2.2. The LS Risk Test

**Figure 1:**
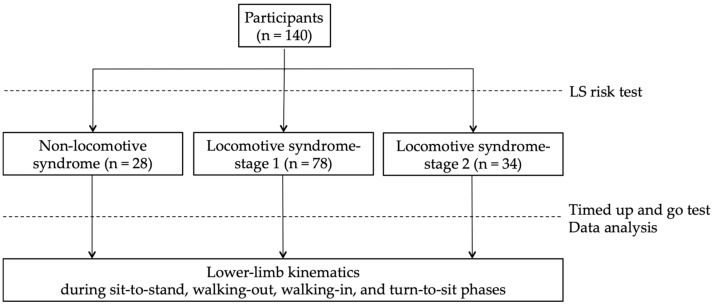
Study design: participants performed the LS risk test to divide them into the non-LS, LS-stage 1, and LS-stage 2 groups and performed the timed up and go test.

The participants performed the LS risk test to divide them into the non-LS, LS-stage 1, and LS-stage 2 groups (Figure 1). The LS risk test, performed following the JOA guidelines, consists of three parts: the two-step test, the stand-up test, and the 25-question Geriatric Locomotive Function Scale (GLFS-25). In the two-step test, the length of two steps from the starting line to the tip of the toes was measured [22]. The score was calculated by normalizing the maximum length of the two steps by height. In the stand-up test, the ability to stand on one or both legs from a 40 cm, 30 cm, 20 cm, or 10 cm high seat was measured [23]. Results are reported as the height of the smallest seat from which the participant could stand. The GLFS-25 is a comprehensive self-report questionnaire that refers to the previous month [24]. The scale includes 4 questions on pain, 16 questions on the activity of daily living, 3 questions on social functioning, and 2 questions on mental health status. Each question is rated from no disability (zero points) to severe disability (four points). LS-stage 1 is defined as a score of less than 1.3 on the two-step test, having difficulty standing up on one leg (or either leg) from a 40 cm high seat, or a GLFS-25 score of seven or higher. LS-stage 2 is defined as a score of less than 1.1 on the two-step test, having difficulty standing up from a 20 cm high seat using both legs on the stand-up test, or a GLFS-25 score of 16 or higher. In this study, the participants who met these criteria for LS risk stage 1 or 2 were defined as the LS-stage 1 or LS-stage 2 groups, and other participants were defined as the non-LS group [25].

### 2.3. TUG Test Using the Motion Analysis System

**Figure 2:**
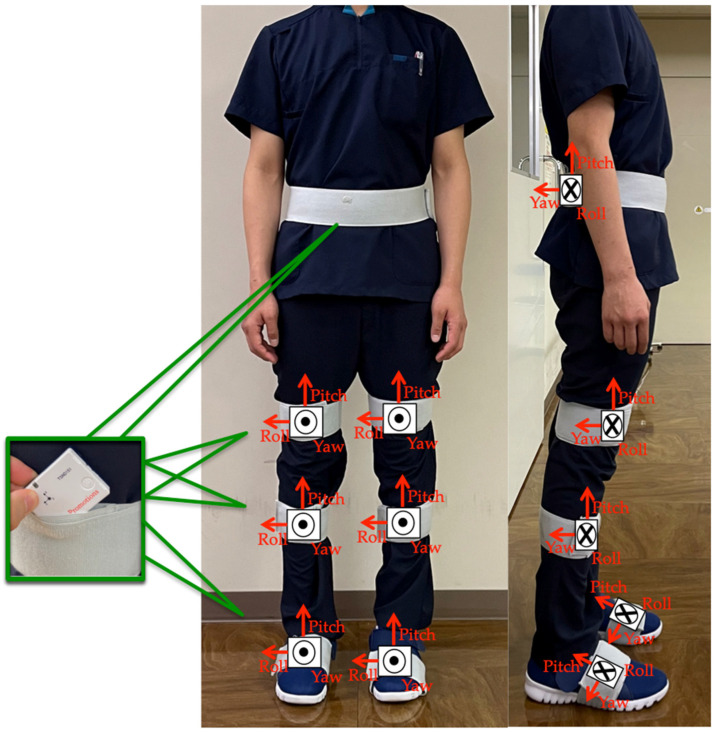
Sensor placements: sensor units were attached to six body segments of the lower limbs and to the pelvis.

The participants performed the TUG test while wearing seven wearable sensor units (TSDN121, ATR-Promotions, Inc., Kyoto, Japan). These sensors consisted of tri-axial acceleration and gyro sensors and were placed on seven lower-limb body segments (pelvis, right and left thighs, right and left shanks, and right and left feet) (Figure 2). Data were collected using a motion analysis system (H-Gait system, Laboratory of Biomechanical Design, Hokkaido University, Sapporo, Japan) where wearable sensors analyzed the lower-limb kinematics [7,17].

A standard armless chair (height: 45 cm) was placed, and the point of return was indicated on the floor 3 m away from it. To enable the motion start determination by the H-Gait system, the participants held a sitting position for about 10 s before performing the TUG test. To clarify the endpoint of the TUG, the participants were instructed to hold the sitting posture for about three seconds after sitting and then return to the static standing posture. In the TUG test, the participants were instructed to stand up, walk to the point of return, turn 180°, walk back to the chair, turn 180°, and sit down again. The participants were instructed to walk at their maximum walking speed for all trials. Each participant was allowed one familiarization trial, and two main trials were performed. The trial with the shorter TUG completion time was used for analysis.

### 2.4. Data Analysis Using the H-Gait System

**Figure 3:**
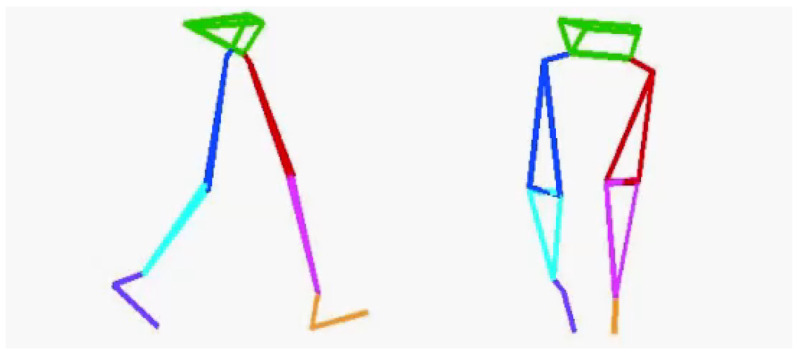
A wire-framed model was used to quantify the lower limb posture during gait.

**Figure 4:**
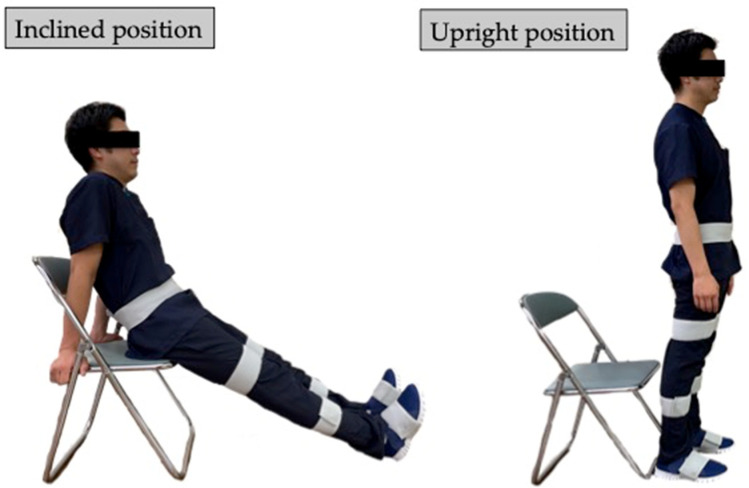
Subject positions for sensor calibration. Sensor calibration was computed from measurements in the inclined and upright positions.

Before the TUG test, accurate lower-limb segment measurements and initial sensor attachment positions must be established. Ten spherical foam polystyrene markers with a diameter of 2 cm were attached to the greater trochanters, medial and lateral femoral epicondyles, and medial and lateral malleoli. Three still images were taken from the right, front, and left sides of the participant in sequence using a digital camera (EX-F1, CASIO COMPUTER Co., Ltd., Tokyo, Japan). Subsequently, the distance between the right and left greater trochanters, thigh length (from the greater trochanter to the lateral femoral condyle), length of the lower leg (from the lateral femoral condyle to the lateral malleolus), and foot height (from the lateral malleolus to the floor) were measured with a ruler. Based on these measurements, the H-Gait system used the wire-framed human gait model to quantify the lower-limb posture during the TUG test (Figure 3). Additionally, sensor calibration was performed for each participant in the upright and inclined positions to calculate the initial inclination of each sensor before the TUG test (Figure 4). This calibration method measures the acceleration data of the sensors in the upright and inclined positions and calculates the initial inclination of each sensor relative to gravity. We assumed that the wearable sensors were placed in a 2-D sagittal plane and derived a rotation matrix from transforming the sensor coordinate system measurements into a global coordinate system. This implementation minimized the effect of attachment errors on the wearable sensors located in front of the segments.

Data analysis was performed using MATLAB software (Math Works Inc., Natick, MA, USA) with a customized motion analysis program. Acceleration and angular velocity data were recorded during the TUG test via a wireless connection in real-time at a sampling rate of 100 Hz and post-processed. The acceleration measured during the upright phase was used to measure the inclination angle of each sensor with respect to the gravitational acceleration direction. This is because a tri-axial acceleration sensor can measure gravitational acceleration, and during a static phase, the orientation of a sensor can be estimated:

(1) θi=cos−1aig

where θ is the inclination angle, *a* is the acceleration value acquired from the sensors, and *i* are the axes *x*, *y*, and *z*.

The drift removal protocol introduced by Takeda et al. [26] was implemented. This involved several protocols: The first protocol, (a), was a calibration procedure for decreasing the attachment errors of the sensors by measuring the gravitational acceleration vector for each sensor in a standing and sitting posture. A rotation matrix was derived from these two measures of the gravity vector allowing for sensor axial measurements to be converted to global coordinate measurements. The second protocol, (b), was done by removing the offset values of the angular velocity data by subtracting the mode value of each sensor and each axis during a static state. The final protocol, (c), was done through digital filtering. This involved applying a 4th-order Butterworth low pass filter with a cutoff frequency of 12 Hz to the raw gyro sensor data. Furthermore, a novel double derivative and integration method was used to remove any drift error that linearly increased over time.

After implementing protocols (a), (b), and (c), the angular displacement from the initial upright position was calculated using a quaternion-based expression [27]. Three-dimensional rotation can be expressed as follows:

(2) $$q=\cos\frac{\varphi }{2}+n\sin\frac{\varphi }{2}$$

(3) $$r’=qrq^{*}$$

where *q* is the quaternion, and *φ* the rotation of angle around unit vector ***n***. q^*^ is the conjugated quaternion, and ***r’*** is the vector after a rotation is applied to the ordinary vector ***r***. The rotation around a given axis is calculated from the angular velocity data and is defined as:

(4) φ=||ω||Δt

where ***ω*** is the angular velocity vector at time *Δt*. The angular displacement can therefore be calculated by continuously calculating *q* for every sampling time point.

### 2.5. TUG Subtasks

**Figure 5:**
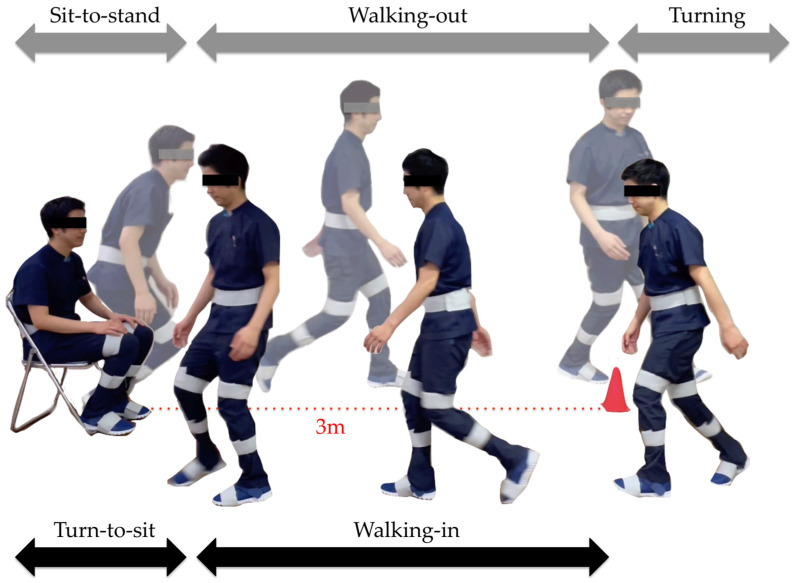
Subtasks: sit-to-stand, walking-out, turning, walking-in, and turn-to-sit phases.

**Figure 6:**
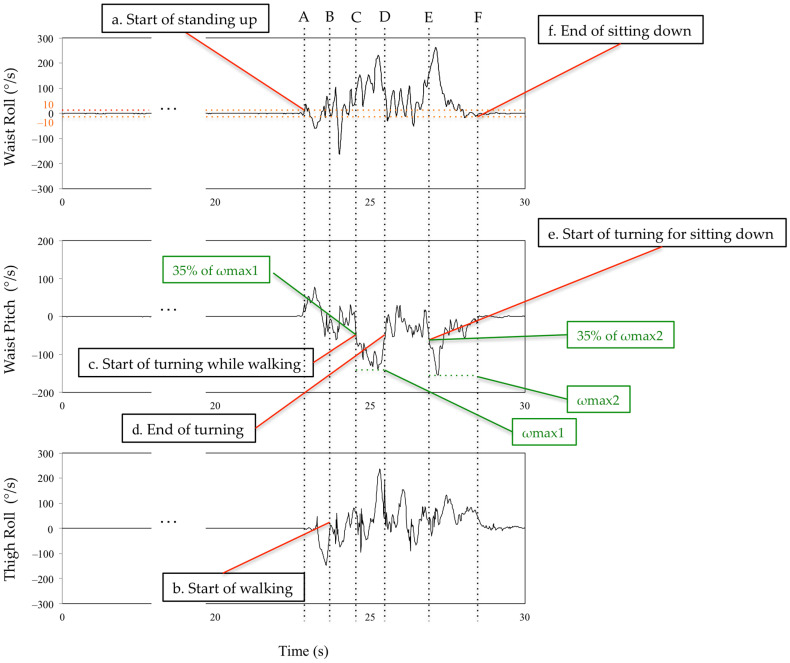
Definition of the limits of the subtasks using the waist and thigh sensors. See main text for details. A,B: sit-to-stand phase. B,C: walking-out phase. C,D: turning phase. D,E: walking-in phase. E,F: turn-to-sit phase.

The subtask identification classifies subtasks into five classes: sit-to-stand, walking-out, turning, walking-in, and turn-to-sit phases [28] (Figure 5). Waist and thigh gyro sensors were used for this classification [29] (Figure 6). During the sit-to-stand phase, the start of standing up was defined as the output of the waist gyro sensor in the roll direction exceeding 10°/s (Figure 6: a). Concerning the walking-out phase, the start of the walking-out phase was defined as the output of the thigh gyro sensor in the roll direction exceeding 10°/s (Figure 6: b). The end of the walking-out phase was defined as the point at which the waist gyro sensor in the roll direction exceeded 35% of its regional maximum absolute value (Figure 6: c), and the total walking-out phase was defined as b, c in Figure 6. The regional maximum absolute value after the start of walking was defined empirically (Figure 6: ωmax1) because the walking-out distance is only 3 m. Therefore, the start of the walking-out phase and the start of the turning phase occur in a short interval of time. Considering the turning phase (Figure 6: c, d), the start of the turning phase was defined as the same time as the end of the walking-out phase, and the end of the turning phase was defined as the point where the angular velocity is below 35% of the maximum angular velocity of the waist gyro sensor in the roll direction (Figure 6: d). Concerning the walking-in phase (Figure 6: d, e), the start of the walking-in phase was defined as the same time as the end of the turning phase, and the end of the walking-in phase was defined as the point where the angular velocity exceeded 35% of the regional maximum angular velocity of the waist gyro sensor in the roll direction (Figure 6: e). The regional maximum absolute angular velocity of the waist gyro sensor in the roll direction was empirically calculated considering the obvious fact that the start of turning for sitting down takes place before the end of sitting down (Figure 6: ωmax2). The end of the turn-to-sit phase occurred when the roll angular velocity in the waist gyro sensor was below 10°/s (Figure 6: f), and the turn-to-sit phase was defined as e, f in Figure 6. TUG completion time was calculated as the interval from a–f in Figure 6.

**Figure 7:**
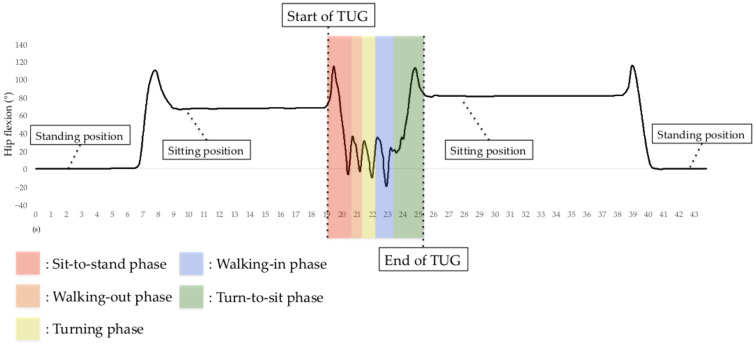
Hip flexion angle before, during, and after the TUG test using the H-Gait system. In the TUG test using the H-Gait system, the participants were in a static standing position before and after the TUG test.

In addition to measuring TUG test completion time, we measured the time for all TUG subtasks. The H-Gait system generates lower-limb kinematics on both lower limbs for all TUG subtasks except for the turning phase. The H-Gait system was developed for walking in a straight line. We failed to evaluate lower-limb kinematics for the turning phase since our drift removal method is not available for the turning phase. Therefore, in this study, only four phases were analyzed, excluding the turning phase. Figure 7 shows the hip flexion angle during the TUG test.

**Figure 8:**
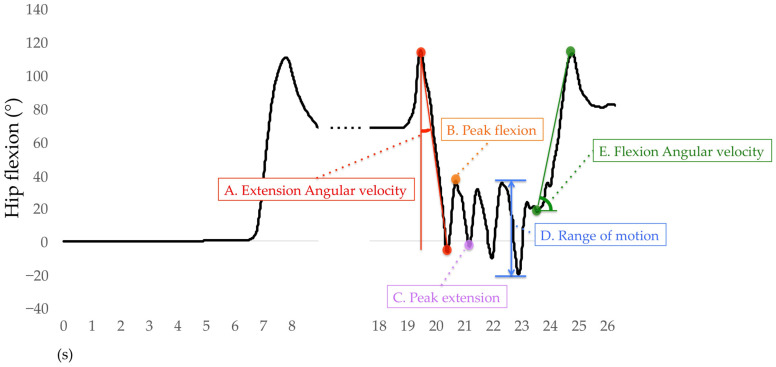
Definition of variables for hip flexion analysis.

Only the right lower limb results are used for analysis. For the sit-to-stand phase, the average angular velocities of hip extension, knee extension, and ankle plantar flexion were calculated. The average angular velocity of the hip extension was the difference between the peak hip flexion to the peak hip extension during the sit-to-stand phase divided by time (Figure 8A) and also for the knee and ankle joints. During the walking-out phase, the mean peak angles of hip flexion, hip extension, knee flexion, knee extension, ankle dorsiflexion, and ankle plantar flexion were calculated. The H-Gait system divided the TUG test into gait cycles and calculated the angles of each joint for every gait cycle. One gait cycle was defined from the heel contact to the next heel contact was normalized to 100%. The heel contact timings were detected using the peak angular velocity data of the shank. The mean peak flexion and extension angles of the hip, knee, and ankle joints for each gait cycle are shown in Figure 8B,C. If two or more gait cycles occurred during the walking-out phase, these peak angles were averaged and retained for analysis. The mean range of motion (ROM) of the hip, knee, and ankle joints was calculated during the walking-in phase. The ROM was defined as the difference between the maximum and minimum flexion angles of each joint. The hip ROM for each gait cycle was calculated during the walking-in phase, as shown in Figure 8D, as well as for the knee and ankle joints. For the turn-to-sit phase, the average angular velocities of hip flexion, knee flexion, and ankle dorsiflexion were calculated. The average angular velocity of hip flexion was the displacement of the peak hip extension to the peak hip flexion during the turn-to-sit phase divided by time (Figure 8E) and also for the knee and ankle joints.

### 2.6. Statistical Analysis

One-way analysis of variance (ANOVA) was conducted to compare demographic characteristics, TUG completion times, duration of TUG subtasks, and lower-limb kinematics during the TUG test among the groups. Differences in sex ratios among the groups were tested with the chi-squared test. The Tukey HSD test was used for post hoc pairwise comparisons. The level of significance was set as α = 0.05. All statistical analyses were performed using SPSS Statistics 22 (IBM Corporation, Armonk, NY, USA).

## 3. Results

### 3.1. Demography, TUG Completion Time, and Duration of TUG Subtasks

The group classification of the participants in this study was 28 in the non-LS group, 78 in the LS-stage 1 group, and 34 in the LS-stage 2 group. One-way ANOVA showed significant differences among the groups in age (*p* = 0.012) and the TUG completion time (*p* < 0.001) (Table 1). The non-LS group was significantly younger than the LS-stage 1 (*p* = 0.015) and LS-stage 2 groups (*p* = 0.026). The TUG completion time in the LS-stage 2 group was significantly greater than in the non-LS (*p* < 0.001) and the LS-stage 1 (*p* < 0.001) groups, and that in the LS-stage 1 group was significantly greater than in the non-LS group (*p* = 0.004). There were no significant differences among the three groups in the other parameters. Additionally, there were no significant differences among the three groups in terms of the duration of TUG subtasks (Table 2).

### 3.2. Lower-Limb Kinematics during the TUG Test

During the sit-to-stand phase, one-way ANOVA showed significant differences among the groups in angular velocities of hip extension (*p* < 0.001) and knee extension (*p* < 0.001) (Table 3). Angular velocities of hip extension and knee extension in the LS-stage 2 group were significantly smaller than in the non-LS (hip: *p* < 0.001; knee: *p* < 0.001) or the LS-stage 1 (hip: *p* < 0.001; knee: *p* = 0.001) groups. Those in the LS-stage 1 group were significantly smaller than in the non-LS group (hip: *p* = 0.016; knee: *p* = 0.025). There was no difference among the groups in the ankle plantar flexion angular velocity during the sit-to-stand phase.

For the walking-out phase, significant differences were found among the groups in the peak flexion angle of the hip (*p* = 0.045) and knee (*p* = 0.002) joints and the peak extension angle of the hip joint (*p* = 0.019). The peak flexion and extension angles of the hip joint were significantly smaller in the LS-stage 2 group than in the non-LS group (flexion: *p* = 0.035; extension: *p* = 0.030). Additionally, the peak flexion angle of the knee joint was significantly smaller in the LS-stage 2 group than in the non-LS (*p* = 0.030) and the LS-stage 1 (*p* = 0.038) groups. There were no other differences among the groups in the peak joint angles during the walking-out phase.

During the walking-in phase, one-way ANOVA showed significant differences among the groups in the knee ROM (*p* = 0.027). The knee ROM was significantly smaller in the LS-stage 2 group than in the non-LS group (*p* = 0.023). There were no other differences among the groups in the ROM during the walking-in phase.

During the turn-to-sit phase, one-way ANOVA showed significant differences among the groups in the hip flexion angular velocity (*p* = 0.012). The hip flexion angular velocity was significantly smaller in the LS-stage 2 group than in the non-LS (*p* = 0.021) and the LS-stage 1 (*p* = 0.024) groups. There were no other differences among the groups in the angular velocities during the turn-to-sit phase.

## 4. Discussion

This study was the first to evaluate the characteristics of lower-limb kinematics during the TUG test in subjects with LS using the H-Gait system. In particular, the angular velocity of the hip and knee extension during the sit-to-stand phase in the LS-stage 1 group was significantly smaller than in the non-LS group. The differences between the non-LS and LS-stage 1 groups were difficult to detect during the 10 m walking test [9]. Additionally, angular velocities of the hip extension and knee extension during the sit-to-stand phase in the LS-stage 2 group were significantly smaller than in the non-LS or LS-stage 1 groups. The ability to stand up from a chair is necessary for the elderly to maintain an independent lifestyle because it is an activity that is performed before walking and other basic activities of daily living. Since the sit-to-stand movement goes from a stable position to an unstable position, it requires greater muscular effort and joint ROM, making it particularly difficult for the elderly to stand up quickly [30]. In addition, the sit-to-stand movement in the elderly becomes slower with aging due to a decrease in muscular function related to the extension of the hip joint [31]. Concerning the decrease in the hip extension angular velocity during the sit-to-stand phase in the LS-stage 1 and LS-stage 2 groups, the frail elderly need training to reduce muscle weakness related to hip extension [31] and sit-to-stand training has been shown to improve muscle strength [32]. Therefore, this study suggests the need for training that includes sit-to-stand movement to prevent LS-stage 1 in the elderly. On the other hand, the hip flexion angular velocity during the turn-to-sit phase was significantly smaller in the LS-stage 2 group than in the non-LS group and LS-stage 1 group. Stand-to-sit training may also be necessary to prevent progression from LS-stage 1 to LS-stage 2.

The peak flexion and extension angles of the hip joint during the walking-out phase were significantly smaller in the LS-stage 2 group than in the non-LS group in this study. Additionally, the peak flexion angle of the knee joint was significantly smaller in the LS-stage 2 group than in the non-LS and LS-stage 1 groups. These results were similar to our previous study during the 10 m walking test [9]. In the current study, maximum walking speed was used during the TUG test, whereas in this previous study, self-selected speed was used during the 10 m walking test. The results from the two studies suggest that the differences in kinematics among the three groups during walking may be similar regardless of the test method or walking speed.

During the walking-in phase, the knee ROM was significantly smaller in the LS-stage 2 group than in the non-LS group. This occurs because during the walking-in phase, it is necessary to slow down the walking speed and apply braking in the walking-in phase due to the transition to the turn-to-sit movement. At the end of walking, the knee ROM is smaller in the elderly than in middle-aged people [33], suggesting that the knee ROM may be even smaller in the subjects with LS, in which walking ability and muscle strength are more impaired. In particular, a decrease in knee extension moment due to aging has been shown in the elderly [34], indicating that it is necessary to improve knee extension muscular strength to prevent and treat LS. In the future, we should investigate whether the training that improves knee extension muscular strength is effective in preventing and treating LS.

TUG completion time is excellent for evaluating waking ability and gait function, and the subjects with LS have been shown to have a longer TUG completion time than the subjects with non-LS [13]. In this study, the TUG completion time was greater in the LS-stage 2 group than that in the non-LS and LS-stage 1 groups, and the LS-stage 1 group’s completion time was significantly greater than in the non-LS group. However, walking ability may not be significantly decreased in the LS-stage 1 and LS-stage 2 groups. This is because the normal value of TUG completion time in the elderly is less than 10 s, which indicates no problem with walking ability [11,35]. The LS-stage 2 group in this study had a TUG completion time of fewer than 10 s, except for one participant, indicating that there were no problems with walking ability. Additionally, there were no significant differences among the three groups in terms of the duration of the TUG subtask in this study. Therefore, the evaluation of lower-limb kinematics during the TUG test, as in this study, is appropriate for the detection of LS.

With the rapid aging of the population, the prevention of LS is an urgent issue. Since the LS risk test is a semi-quantitative and comprehensive assessment, a more detailed assessment was needed to evaluate the walking ability in detail to detect early signs and prevent LS. Therefore, it may be useful to evaluate gait characteristics using a wearable sensor-based motion analysis system for its early detection and prevention. We have shown that the wearable sensor-based motion analysis system, the H-Gait system, can perform large-scale gait analysis during the TUG test in a short time regardless of location. In particular, our findings indicate that evaluation of the lower-limb kinematics during the TUG test using the H-Gait system is highly sensitive to detect LS-stage 1, compared with evaluation of the lower-limb kinematics during the 10 m walking test. The TUG test using the H-Gait system for residents in various local communities will enable early detection of LS and will lead to its prevention or treatment. The TUG test using the H-Gait system can routinely and more flexibly assess LS risk and can make a significant contribution to LS risk detection among community-dwelling elderly. If abnormal kinematics are found during the TUG test, treatment can be given to each individual to prevent LS. Therefore, our findings provide useful evidence for the primary and secondary prevention of LS and will contribute greatly to the increase in healthy life expectancy. In future studies, we believe that prospectively evaluating the gait characteristics of the elderly using the H-Gait system will help to identify lower-limb kinematics associated with the progression of LS.

This study has several limitations. First, only local residents who utilized the health promotion facility were included in this study. It was not clear whether the results of this study would apply to other local residents who did not utilize the health promotion facility. Second, because this study was a cross-sectional study, the causal relationship between the onset of LS and differences in the lower-limb kinematics during the TUG test was unknown. Third, sample size differences among the three groups may have affected the results. Finally, the lower-limb kinematics during the turning phase could not be evaluated because the H-gait system does not allow for programs that include a turning phase.

## 5. Conclusions

This study evaluated the characteristics of lower-limb kinematics during the TUG test in non-LS, LS-stage 1, and LS-stage 2 subjects using the wearable sensor-based H-Gait system. Compared with the non-LS group, the LS-stage 1 and LS-stage 2 groups showed significantly smaller angular velocity of hip and knee extension during the sit-to-stand phase. The LS-stage 2 group showed significantly smaller peak angles of hip extension and flexion during the walking-out phase compared to the non-LS group. These findings indicate that the evaluation of the lower-limb kinematics during the TUG test using the H-Gait system is highly sensitive to detect LS, compared with the evaluation of lower-limb kinematics during the 10 m walking test.

## Figures and Tables

**Table 1:** Demography and TUG completion time. Statistical results from one-way ANOVA for group comparison.

	Non-LS (*n* = 28)	LS-Stage 1 (*n* = 78)	LS-Stage 2 (*n* = 34)	*p* Value
Age (years)	69.3 (5.3)	73.4 (7.0) *	73.7 (6.7) *	0.012
Gender (men/women)	4/24	15/63	3/30	0.375
Height (cm)	151.5 (7.2)	153.9 (7.2)	150.8 (7.1)	0.072
Mass (kg)	50.7 (8.0)	54.3 (7.4)	53.7 (10.5)	0.162
TUG completion time (s)	5.5 (0.9)	6.4 (1.2) *	7.4 (1.6) *^,#^	<0.001

Data are presented as mean (standard deviation). LS: Locomotive syndrome, TUG: Timed up and go. * *p* < 0.05 (vs. the non-LS), ^#^ *p* < 0.05 (vs. the LS-stage 1).

**Table 2:** Duration of TUG subtasks. Statistical results from one-way ANOVA for group comparison.

Subtasks	Non-LS (*n* = 28)	LS-Stage 1 (*n* = 78)	LS-Stage 2 (*n* = 34)	*p* Value
Sit-to-stand (s)	0.5 (0.2)	0.6 (0.2)	0.7 (0.3)	0.210
Walking-out (s)	1.3 (0.4)	1.5 (0.4)	1.6 (0.4)	0.467
Turning (s)	0.8 (0.2)	1.4 (0.6)	1.6 (0.9)	0.098
Walking-in (s)	0.7 (0.2)	1.0 (0.3)	1.2 (0.8)	0.087
Turn-to-sit (s)	1.3 (0.4)	2.1 (0.8)	2.2 (0.9)	0.251

Data are presented as mean (standard deviation). LS: Locomotive syndrome.

**Table 3:** Lower-limb kinematics. Statistical results from one-way ANOVA for group comparison.

Subtasks	Kinematics	Non-LS (*n* = 28)	LS-Stage 1 (*n* = 78)	LS-Stage 2 (*n* = 34)	*p* Value
Sit-to-stand	Hip extension angular velocity (°/s)	134.0 (33.4)	116.3 (29.1) *	91.7 (23.7) *^,#^	<0.001
	Knee extension angular velocity (°/s)	113.3 (31.0)	96.8 (29.9) *	75.5 (21.7) *^,#^	<0.001
	Ankle plantar flexion angular velocity (°/s)	76.8 (46.7)	72.2 (53.7)	74.8 (79.0)	0.421
Walking-out	Peak hip flexion (°)	43.1 (10.8)	39.8 (10.9)	36.5 (8.4) *	0.045
	Peak hip extension (°)	1.9 (7.6)	0.9 (6.6)	−2.6 (6.7) *	0.019
	Peak knee flexion (°)	76.6 (13.5)	69.9 (16.4)	61.8 (16.7) *^,#^	0.002
	Peak knee extension (°)	2.0 (2.6)	2.0 (2.9)	2.2 (2.4)	0.704
	Peak ankle dorsiflexion (°)	13.7 (11.2)	13.6 (11.7)	12.7 (13.1)	0.918
	Peak ankle plantar flexion (°)	15.5 (11.8)	14.9 (11.1)	16.8 (12.3)	0.711
Walking-in	Hip range of motion (°)	46.3 (15.5)	42.2 (13.7)	36.8 (12.8)	0.051
	Knee range of motion (°)	59.2 (13.6)	54.4 (12.7)	50.8 (14.0) *	0.027
	Ankle range of motion (°)	30.4 (20.4)	29.0 (14.8)	30.2 (21.3)	0.908
Turn-to-sit	Hip flexion angular velocity (°/s)	97.5 (19.5)	94.1 (23.6)	81.5 (25.4) *^,#^	0.012
	Knee flexion angular velocity (°/s)	82.9 (28.8)	79.0 (37.2)	71.1 (29.7)	0.364
	Ankle dorsiflexion angular velocity (°/s)	76.8 (46.7)	72.2 (53.7)	74.9 (59.2)	0.935

Data are presented as mean (standard deviation). LS: Locomotive syndrome. * *p* < 0.05 (vs. the non-LS), ^#^ *p* < 0.05 (vs. the LS-stage 1).

## Data Availability

The datasets used and/or analyzed during the current study are available from the corresponding author upon reasonable request.
